# Ethical and legal considerations influencing human involvement in the implementation of artificial intelligence in a clinical pathway: A multi-stakeholder perspective

**DOI:** 10.3389/fdgth.2023.1139210

**Published:** 2023-03-13

**Authors:** Elizabeth Redrup Hill, Colin Mitchell, Tanya Brigden, Alison Hall

**Affiliations:** PHG Foundation, University of Cambridge, Cambridge, United Kingdom

**Keywords:** artificial intelligence, medical ethics, law, digital pathology, healthcare, interdisciplinary, human involvement

## Abstract

**Introduction:**

Ethical and legal factors will have an important bearing on when and whether automation is appropriate in healthcare. There is a developing literature on the ethics of artificial intelligence (AI) in health, including specific legal or regulatory questions such as whether there is a right to an explanation of AI decision-making. However, there has been limited consideration of the specific ethical and legal factors that influence when, and in what form, human involvement may be required in the implementation of AI in a clinical pathway, and the views of the wide range of stakeholders involved. To address this question, we chose the exemplar of the pathway for the early detection of Barrett's Oesophagus (BE) and oesophageal adenocarcinoma, where Gehrung and colleagues have developed a “semi-automated”, deep-learning system to analyse samples from the Cytosponge^TM^ TFF3 test (a minimally invasive alternative to endoscopy), where AI promises to mitigate increasing demands for pathologists' time and input.

**Methods:**

We gathered a multidisciplinary group of stakeholders, including developers, patients, healthcare professionals and regulators, to obtain their perspectives on the ethical and legal issues that may arise using this exemplar.

**Results:**

The findings are grouped under six general themes: risk and potential harms; impacts on human experts; equity and bias; transparency and oversight; patient information and choice; accountability, moral responsibility and liability for error. Within these themes, a range of subtle and context-specific elements emerged, highlighting the importance of pre-implementation, interdisciplinary discussions and appreciation of pathway specific considerations.

**Discussion:**

To evaluate these findings, we draw on the well-established principles of biomedical ethics identified by Beauchamp and Childress as a lens through which to view these results and their implications for personalised medicine. Our findings are not only relevant to this context but have implications for AI in digital pathology and healthcare more broadly.

## 1. Introduction

Artificial Intelligence (AI) promises to improve the efficiency and accuracy of many aspects of healthcare. However, clinical efficiency and algorithmic accuracy are not necessarily synonymous (1), and consequently, consideration of when, if at all, AI should replace human experts in healthcare pathways is increasingly important. One approach to this question focuses largely on the performance of the AI tool and how it compares with humans (2), suggesting automation only where performance meets or exceeds that of humans. This stage has been reached in some areas of healthcare and there is a burgeoning literature focused on the technical performance of AI tools (2). However, there is also a growing body of literature which seeks to move beyond the “technology-centric” approach to view the AI tool in context, as part of a wider view of the overall system, taking into account the impact on and responses of human experts and patients (3). This “systems perspective” (3) is also valuable in assessing the ethical and legal impacts of hybrid healthcare pathways because it moves beyond technical accuracy and efficacy, to consider the real-world impact on the humans working with AI and those being assessed and treated. In doing so, it may helpfully indicate design imperatives for the system to maximise human benefit and minimise harm.

A range of general ethical and legal considerations have already been identified as relevant to the adoption of AI in healthcare, including the potential for automation bias, the importance of informed consent, safeguarding against harm and the challenge of ensuring adequate transparency (4–8) Automation bias can lead to discrimination and inequity where, for example, AI has been trained on data that is not representative of minority ethnic groups. Informed consent is also relevant because of how AI can impact patients' decision-making and consequently their legal protections, leading to questions about what amounts to informed consent in specific pathways or contexts. Transparency and oversight are important as such safeguards can help foster trust in the system and ensure that errors are addressed and mitigated where possible.

However, to successfully adopt AI-driven tools in practice requires an understanding of what a full range of stakeholders including patients, healthcare professionals, regulators and policymakers consider to be the key ethical and legal considerations, and how they apply in a specific healthcare pathway or context.

We aimed to develop evidence on the ethical and legal factors that are identified in a specific application of AI in a digital pathology context, through interdisciplinary discussion between developers, primary users (clinicians), wider healthcare patient representatives, regulators, and policymakers. In this paper we identify the factors that these stakeholders consider to be important, areas of agreement and areas of disagreement, and implications for design and policy for the implementation of AI in healthcare. We also highlight the importance of an open dialogue between interdisciplinary teams to delineate what the legal and ethical needs are and how they influence, when, and in what form, human involvement may be required in the context of a specific clinical pathway.

To focus stakeholder discussions, we used the exemplar of the pathway for the early detection of Barrett's Oesophagus (BE) and oesophageal adenocarcinoma, for which Gehrung et al. have developed a “semi-automated”, deep-learning system to analyse samples from the Cytosponge^TM^-TFF3 test (a minimally invasive alternative to endoscopy) (9). The Cytosponge^TM^-TFF3 test utilises a sponge on a string which the patient swallows to collect cellular samples from the length of the oesophagus. Unlike other sampling methods, such as endoscopic biopsy, the cells collected from the Cytosponge^TM^ are a pan-oesophageal sample of different cell types which does not maintain the tissue architecture. Laboratory processing concentrates the cells collected by the sponge which are then formalin fixed and embedded in paraffin wax. Very thin sections are cut and stained with a hematoxylin and eosin stain (for quality control) and TFF3 (trefoil factor 3) which is a key diagnostic biomarker of BE enabling the identification and quantification of goblet cells (9). These stained slides are then scanned and digitized. During the training process, digitized slides were annotated and reviewed by an experienced pathologist using specific software and tessellated to prepare for model training. More information on these processes are available in Gehrung and colleagues' papers, see endnote (9). Through this combination of bespoke stains and digital pathology, the Cytosponge^TM^-TFF3 test aims to identify cellular changes which could indicate areas of Barrett's oesophagus or other focal areas of intestinal metaplasia.

This application provides a useful exemplar of how AI could help pathologists deal with unconventional sample types (the Cytosponge^TM^), and reduce the time taken to report that large cell sample (1–4 M cells are retrieved), as well as meeting the consequent increased screening demand for pathologists. These findings are not only relevant to this context but have implications for AI in digital pathology and healthcare more broadly.

## 2. Methods

We convened four virtual workshops with key stakeholders (*n* = 31) to obtain their insights and views on the following question: what ethical and legal factors influence the nature and level of human involvement that is necessary or desirable in AI-driven systems for digital pathology and healthcare? Potential stakeholders were based on their expertise in a relevant area, based on their knowledge of Project DELTA, or as representatives of a particular stakeholder group (e.g., disease associations). The stakeholders included, software developers (*n* = 4) and pathologists (*n* = 7) (workshop one), professional body representatives and policy, legal and ethical experts (*n* = 11) (workshop two), and representatives from relevant patient groups or charities and frontline healthcare professionals (*n* = 9) (workshop three). The findings were presented collectively to all stakeholders for further comment in a plenary workshop. The workshops were recorded for the purposes of note taking only and consent was obtained from all stakeholders. We grouped the discussion points into key ethical and legal issues for further analysis. After identifying these issues, we evaluated these against the well-established biomedical ethical benchmark of Beauchamp and Childress's Four Principles (10). This framework is familiar to most health care professionals forming part of the curriculum in many medical courses. The synergies and differences between the findings from the workshop, and this framework, help to illustrate some potential implementation challenges in this area.

## 3. Results

The stakeholders identified a wide range of ethical and legal considerations that are relevant in determining the nature and level of human involvement that may be appropriate in the implementation of AI tools along a diagnostic pathway. Some of these are relevant to many areas of medicine, whilst others may be a greater priority in digital pathology. Different stakeholder groups also frequently identified similar themes but from different perspectives and with different prioritisation of how pressing they are. Using the semi-automated approach developed by Gehrung et al., as an exemplar, helped to draw out more granular considerations and greater nuance within the broader ethical and legal themes. The key ethical and legal considerations can be grouped under six general themes. In this section we outline these themes with some detailed examples. In the subsequent discussion we reflect on the implication of these findings for design, regulation and implementation of AI in digital pathology and healthcare more widely.

### 3.1. Risks and potential Harms

All stakeholders raised risks and potential harms as relevant to determining the level of human involvement/automation in a clinical pathway. The commonly-known issue of black box algorithms and the difficulty of auditing them where harm occurs was raised. It was acknowledged that regulatory safeguards may help mitigate this risk through the requirement of rigorous evidential demands before blackbox AI can be implemented in a real-world context. In turn, this also raised the counter-consideration of AI exceptionalism, which questions whether ethical and legal approaches to AI demand higher standards than we currently demand of humans, and consequently impedes the realisation of the necessary benefits that AI has to offer to the detriment of human experts and patients. We found that the risks of not automating could be subcategorised into risks of exceptionalism and the potential harms that might result.

In relation to harm, the participants considered the overstretched NHS, the rising demand for pathology expertise and the need to improve diagnostic performance where AI can help to reduce workload and resource burdens resulting from low-risk tasks, leaving human experts to focus on tackling tricker cases and tasks.

*“We need to seriously consider the risks if AI is not used. Humans should not always stay in the loop. If we have a health system that is over capacity because we’re not using AI that also won’t build trust.”*

They suggested that exceptionalism was one of the greatest potential barriers to the adoption of AI in healthcare, and would likely arise where human expectation sets too high a standard for AI, and might lead to policymakers implementing a draconian regulatory regime. Nevertheless, the UK Government in their response to the Medicines and Healthcare Products Regulatory Agency's (MHRA) consultation has stated that they do not intend to introduce any AI specific requirements in legislation beyond those proposed for software more generally (11).

### 3.2. Impact on human experts

The impact on human experts was a key consideration with a focus on how a human-AI hybrid pathway would impact pathologists and bioinformaticians. Whilst most participants believed that a hybrid pathway could reduce workload and ease personnel pressures, it was still considered imperative that pathologists must continue to oversee the trickiest of cases, such as those involving atypia (particular cellular abnormalities). Therefore, there was a general consensus that AI should not replace pathologists but would shift their role from processors to expert overseers. Participants noted that this potential shift might have a significant impact on the training of junior pathologists, as their expertise is obtained through experience and more specifically, the experience of looking at the whole slide, not only flagged areas.

*“Pathologists will take an active role in overseeing AI. However, they will be left with the trickiest of cases, but this raises the concern of how pathologists will be trained in future if straightforward cases are assessed by AI.”*

Additionally, concerns were also raised that the introduction of AI tools could lead to over-reliance or complacency. Finally, the explainability of AI processed results was discussed and what information the AI should provide to equip professionals with the information necessary to understand the results themselves and explain them to patients.

*“I think GPs and patients will want to know how the AI arrived at that decision. If GPs are not sure why AI reached that decision, there will be concerns that certain models do not provide enough transparency. If the machine tells you, it's positive, it should indicate why, for example, by referring the GP to the areas of the specimen it looked at instead of giving no explanation at all.”*

There was a general consensus that communication of significant test results in areas such as cancer should remain a human role to afford dignity to patients.

### 3.3. Equity and bias

Stakeholders raised the issues of equity and bias, with a focus on how these could be mitigated, and how potential known and unknown biases could be explored. Developers and health care professionals (HCPs) recognised the issue of human bias infiltrating training data sets and debated the under investigation of potential unknown bias in cell imaging. For example, in the context of digital pathology, if a certain form of atypia is shown to hold a link to ethnicity but this has not been used to train the AI, it could lead to unequal treatment for people of underrepresented ethnicities. Ethicists and patient representatives discussed how this could lead to public distrust and potential health inequities. The participants considered whether bias might infiltrate through the training data cohort, where currently those presenting with reflux symptoms are more prevalent. They also discussed how unknown bias could be tackled, reflecting on other healthcare AI that presented discoveries of previously unknown bias. Perception of bias was deemed equally harmful as actual bias due to its potential negative impact on public trust.

*“Perception of bias can be just as damaging. Even if the system is not biased, the lack of a demographic's presence in the training cohort might impact their trust in the system.”*

### 3.4. Transparency and oversight

Stakeholders discussed the importance of human involvement in the form of oversight of the system's decisions and how they have been reached, including how to scrutinise and guard against errors, and how to ensure that HCPs retain ultimate responsibility for decisions made to maintain patient trust and confidence in partially automated pathways.

*“These are statistical and correlational systems; they do not have judgement like doctors. Responsibility is a normative concept; if the human is removed from the loop, it removes the normative dynamic. This must fall on humans in safety critical systems, otherwise, dignity is lost and harm results.”*

Transparency was emphasised as essential for these purposes but this did not lead stakeholders to conclude that HCPs required comprehensive detailed software information about how the system reaches a decision. Instead, the healthcare professionals in our workshop prioritised information which would explain why an AI had flagged specific areas of slides. Other stakeholders agreed on the importance of such information for patients and frontline HCPs, to assist discussions with patients on the implications of their results, for example what the results mean and what the next steps are.

*“Health anxiety is increasing in the digital age. Giving information to allay anxiety is part of the cure. It would be helpful to develop the system with transparency, e.g., saying on the report that this is the diagnostic answer and level of confidence, including options for next steps.”*

The importance of understanding the AI's limitations was also highlighted as important information for frontline clinicians and consequently, it was considered necessary to think about transparency at the design concept stage so that the end report produced by the AI is both interpretable and explainable by HCPs to patients.

### 3.5. Patient information and choice

Stakeholders also highlighted the importance of transparency and its role in earning public trust and confidence in the adoption of AI systems in healthcare. However, there was debate among stakeholders about the level of information and choice that should be provided in relation to AI tools. Pathologists and software developers were wary of an exceptional approach to AI systems, contrasting with other sophisticated technologies, and cautioned against disproportionate requirements for information and choice. For example, the method by which pathology slides are currently processed is not shared with patients and is left to the discretion of HCPs and medical scientists to determine best practices.

*“There is an exceptional treatment of AI. These discussions could apply to a wider range of medical technology and not specifically AI. Do patients need to know extra special information on AI?”*

Ethical and legal experts highlighted that the NHS does not generally operate on a patient choice basis regarding the methods of diagnosis/analysis, and cautioned this could in fact be unethical in such a limited resource health system.

*“The NHS is not designed for choice. The focus on choice may be unethical as more pressing issues exist. We should be cautious of framing this issue around choice.”*

However, it was also considered that it may be unethical if patients who do not trust AI have no other option but to have their results processed by it in future. One notable uncertainty was raised by data protection experts who highlighted that, provisions relating to decisions based on solely automated processing may require explicit consent from patients in certain circumstances. Against this backdrop, the stakeholders considered what information should be provided to patients and the public in general. It was considered that too much information could in fact be detrimental. They considered that information needs to be tailored for HCPs and individual patients. For example, a basic overview of what the AI does and how it reaches a conclusion could be provided with directions to further information for patients who want to find out more.

*“Too much information is unhelpful. Patients would want to know more about how it impacts them and their care options. It matters most when errors occur. Those are moments where transparency really matters to enable patients to seek redress, second opinions and question those decisions. Long-term patients will have great knowledge and will want to question decisions more.”*

It was also suggested that public expectations may need to be managed with appropriate information on the overall nature of the AI system and its real (as opposed to idealised) benefits. For example, pathologists and ethicists noted that it is not guaranteed that AI accuracy is superior to humans as it will only be as good as the humans who train it. Discrepancies between pathologists are commonplace but the idea that AI might also present discrepancies might be interpreted by the public as errors, leading to exceptionalist regulatory treatment and public mistrust. The ethicists and legal experts felt that AI is still fallible and that therefore clarity is needed on the benefits and rationale for introducing AI into healthcare pathways. The participants suggested that its greatest benefit was re-distributing resources in an over-burdened NHS, where it is hoped that this would reduce missed cases and allow more expansive screening of those who are at higher risk of developing BE.

### 3.6. Accountability, moral responsibility and liability for error

A final and important area of ethical and legal discussion related to accountability, moral responsibility and liability for error. Pathologists emphasised an important linguistic difference between discrepancy and error, where discrepancy is common as it is a difference of professional opinion, based on the individual experiences of different pathologists. However, an error is a failure to meet expected standards and therefore should be treated differently in discussions on responsibility and legal liability.

*“Are we talking about discrepancy or error? What clinical impact does a discrepancy have to potential patient harm as opposed to “error?”*

Pathologists assume that liability would only arise where error has occurred, but it remains to be seen how the courts might interpret discrepancy as error due to different disciplinary understandings. This also led to the subsequent consideration of how much discrepancy amounts to error and whether a standard needs to be set for hybrid pathways.

The lack of clarity from regulatory and ethical perspectives on accountability, moral responsibility and legal liability for error were a concern to all participants. All participant groups considered that current regulatory approaches, like those in the UK GDPR, are too general to provide clarity in the context of specific applications in areas like healthcare and are not always translatable to smaller scale AI, like analysing Cytosponge^TM^ slides. For example, some AI can be trained on smaller targeted datasets. Furthermore, current approaches have unanswered questions, such as whether the report that a healthcare AI tool produces amounts to a “decision” for the purposes of the UK GDPR, and whether they engage the restrictions in the UK GDPR against automated individual decision-making, including profiling (12).

Data protection specialists highlighted that such considerations are made relevant by Article 22 of the UK GDPR which prohibits solely automated decision-making which produces “legal effects” or significantly affects the data subject (12, Article 22). These provisions also require additional safeguards for automated processing of health data (12). These restrictions provide for automated processing to proceed in certain cases, where authorised by consent, contract or law, and it is uncertain how compatible these are with nationalised health systems where the legal basis for processing is not based on consent (12).

*“Article 22 is an apparent divergence from practice. The NHS is not designed for choice. If the NHS says no choice this creates a legal tension with Article 22 and its choice for human intervention.”*

Looking ahead to the possibility of greater automation, some HCPs also expressed a wish to understand when and how civil or criminal liability might arise where AI is considered the decision-maker. From a patient perspective, it is also unclear what methods of redress would be available where harm has occurred.

*“It is technically possible to fully automate, but it raises a legal and regulatory question i.e., if a false positive who bears liability? How does that impact access to treatment and how does it deal with the psychological burden it may bear on patients?”*

Finally, stakeholders also considered the issue of negative media portrayals of cognisant AI, leading to the potential for public trust to be undermined. Whilst stakeholders expressed uncertainties, there was agreement that liability, responsibility, and accountability must remain with human experts, including the communication of clinically significant results.

**Figure 1:**
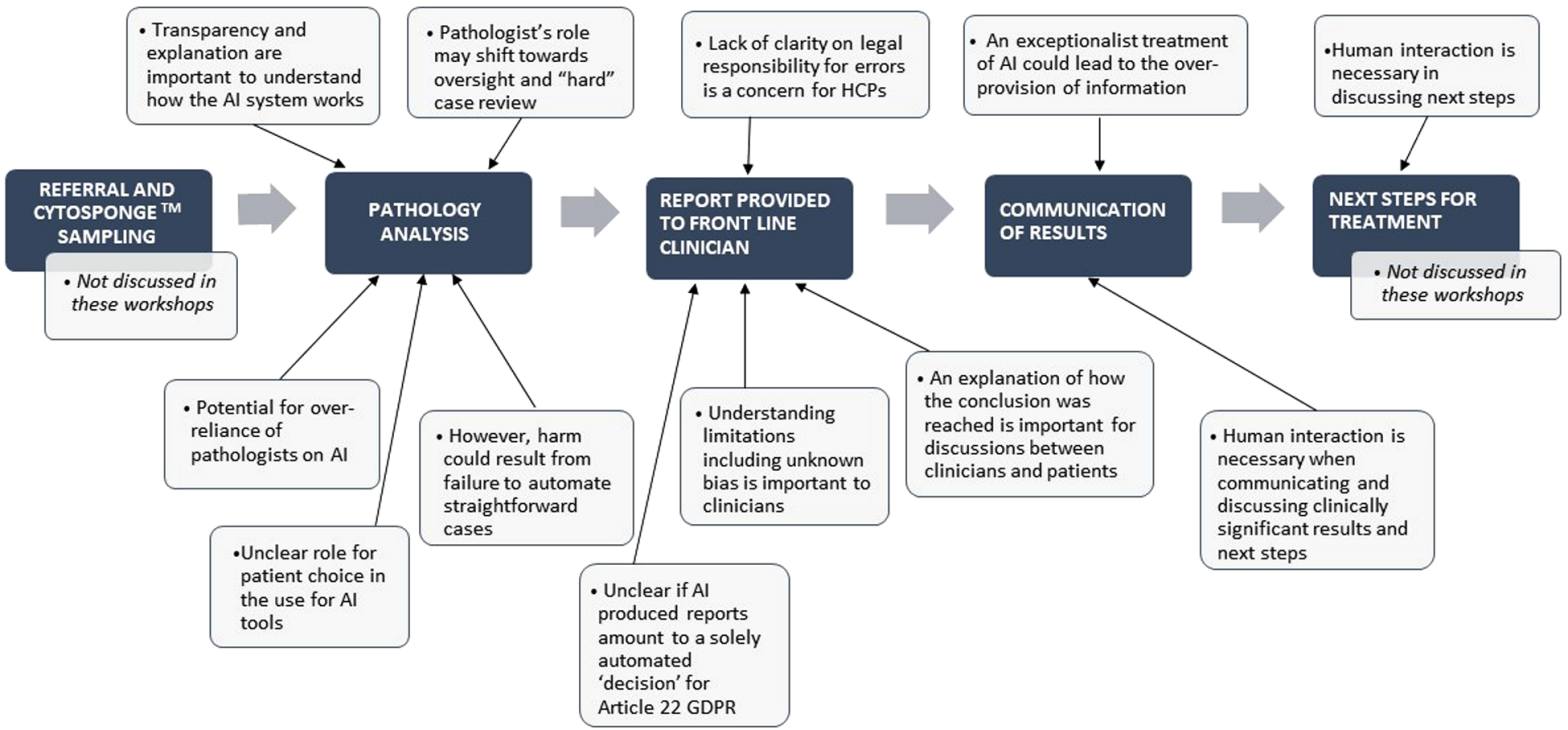
Key ethical and legal considerations in a diagnostic pathway incorporating AI automation.

These ethical and legal considerations are relevant to implementation of AI tools for diagnosis in general. However, some considerations are particularly relevant to certain parts of the diagnostic pathway. Our workshops were focused on three stages within a pathway for diagnosis using Cytosponge^TM^ samples: pathology analysis, provision of the report to frontline clinician and the communication of clinically significant results to the patient. Figure 1 indicates some of the key ethical and legal considerations that relate to specific stages of the pathway, from referral through to treatment and management.

## 4. Discussion

Many of the themes discussed in the workshops will be familiar to those in the AI and health field. However, our findings demonstrate that within these broad themes, a range of subtle and context-specific elements need to be taken into account by developers, HCPs, regulators and policymakers. Stakeholders identified a wide range of ethical and legal factors that may influence the appropriate level and form of human involvement in a particular clinical context. Some may have a more direct impact, such as the requirements of regulation or legal liability, others may be less direct but still ultimately influence the level of automation that is appropriate, such as the importance of maintaining patient and public confidence in the healthcare system.

In order to evaluate these issues further we chose to assess them against the principles of biomedical ethics framework developed by Beauchamp and Childress (10). Practitioners and policymakers are familiar with the principles of biomedical ethics: autonomy, beneficence, non-maleficence and justice to evaluate their practice. We have chosen to utilise this framework as a benchmark for evaluating issues arising in the context of decision-making about AI-driven automation, using it to categorise some of the factors identified by stakeholders in our workshops. This enables a deeper ethical consideration of these factors and what they suggest for decision-making in this area.

### 4.1. Justice and non-maleficence: risk, moral responsibility and legal liability

The ethical principle of non-maleficence (10) is centred on the idea that harming patients is wrong and it therefore plays a key role in helping to determine the risks and avoidable harms of AI-human pathways. Key ethical and legal considerations include the potential risks and harms in the context of specific clinical pathways as well as more widely. To better discover what these are, some have called for a paradigm shift from actively searching for AI's benefits to its weaknesses (2).

Pathologists highlighted that whilst it was important that straight-forward cases be automated, the AI needed to be programmed to refer any atypical cases back to human specialists. Such views also seem to be echoed in research conducted on AI reliability to scan bone fractures where AI struggled with atypical cases/ abnormal bones (13). The workshop participants therefore did not believe it was advisable to have AI assess all cases without human oversight. Human oversight was therefore deemed necessary to prevent harm, particularly in high-risk cases or atypia, but how to define risk comes with its own difficulties. Such assessments therefore evidence the importance of also being aware of the weaknesses of the system.

Stakeholders highlighted the equally important and perhaps underappreciated need to consider that harm also results from not automating necessary elements in a resource-strapped and overburdened environment like the NHS. This potentially places medical decision-makers who over-rely on such tools in a difficult liability position and emphasises the need for medical decision-makers to remember that AI cannot currently replace human expertise and will likely be considered equivalent to a mere diagnostic tool in the short/medium term. Such tools should be used to guide but not determine cases.

Harm arising from both action and omission is reflected in the mirrored ethical principle of nonmaleficence (10). Consequently, participants considered that stakeholders need to be aware that harm could result from not automating just as much as it could from automating. Questions of legal liability and moral responsibility also relate to ethical questions concerning harm and justice. In a publicly funded NHS, resources are limited and consequently there is little room for real patient choice, leading to concerns of inequity or possibly discrimination. Moreover, unrealistic expectations of AI can also lead to public distrust and possible liability claims which raises important considerations for stakeholders on how to avoid not just harming patients, but to avoid undermining trust in this necessary technological development to meet demand and keep patients safe.

Currently, the question of increased automation is treated with trepidation by some developers and HCPs who are concerned with navigating the fast-moving regulatory landscape. In particular, participants noted a potential conflict between the potential need for consent and a choice to be provided under Article 22(1) of the UK GDPR and the realities of a resource-constrained NHS which cannot always provide a choice. Under current healthcare practices patients have limited choice and that choice does not extend to how treatment or diagnosis is conducted i.e., whether their slides are read and processed by humans or AI. However, Article 22 GDPR has brought this practice and policy approach into question as it stipulates that, certain decisions such as those which involve solely automated decisions which produce legal effects or significantly affects the data subject, must provide individuals with the right to have their case reviewed by another human being.

Participants also expressed uncertainty over when AI produced reports will amount to a decision from a legal perspective. The term “AI” may lead to exceptionalist regulatory treatment due to its negative public image. Adopting an exceptionalist approach fails to take account of the fact that there is a spectrum of automation before automation becomes machine cognisance. Nevertheless, although machine cognisance may be a future concern, there is a real current concern that HCP over-reliance will in effect turn AI reports (just one source of evidence) into the decision-makers for diagnosis, i.e., a medical decision. Such debates often turn on whether AI in pathology is merely a diagnostic support tool, much like any other medical test, or whether it amounts to a decision-maker with potentially serious legal effects.

Such considerations also extend to uncertainty felt by developers and HCPs on how AI will align with existing liability frameworks (insurance, tort, criminal, contract etc.). Participants raised questions such as whether insurance companies would provide coverage for HCPs where decisions are being wholly or partly reported by the AI itself. Regulators will need to be both proactive and reactive to the challenges AI presents but responsibility will also fall to the health sector to self-regulate because it is possible that AI will start to set the new gold standard in terms of clinical evidencing standards if it becomes more accurate than human practitioners. Such standards will need to be regularly reviewed by professional regulatory bodies. Therefore, reliance on generalised legislation such as the GDPR or the Data Protection Act 2018 will likely need to be supplemented by sector specific guidance which provide more targeted advice for health care professionals and their patients.

### 4.2. Beneficence: impact on human experts and patients

The ethical principle of beneficence is another of Beauchamp and Childress' widely adopted principles in healthcare (10). The principle is centered on the imperative to do good. This is relevant when considering the implementation of AI and the potential benefits arising. The principle suggests that benefits should not be idealised and that it must be possible to show real world benefits for all human experts and patients along the pathway. Legally, such a principle would be reflected in HCPs' duty of care to their patients.

While much of the literature has focused on AI harm from the perspective of patients, a less discussed perspective was raised by the stakeholders in the workshops in the form of the potential impact on HCPs. The participants predicted that AI will not replace human experts, but instead, that their role will shift to predominantly overseeing automated AI reports on typical cases alongside manual reading of atypical cases. Nevertheless, this raised a subsequent question of how to avoid deskilling pathologists, who will still need to review atypical or complex cases for which expertise can only be gained through experience of both typical and atypical cases. Participants also highlighted that the purported benefit of “time-saving” is difficult to prove because pathologists' time will still be spent elsewhere, suggesting there is a need to map out predicted role shifts in order to answer the fundamental question of how to use AI in an optimal way while easing workload. Therefore, a common point of agreement was that it was not enough to avoid harm, but it was also necessary to demonstrate a positive difference to human experts and patients along the pathway. Determining this “positive difference” would involve a balancing judgement whereby potential benefits outweigh any potential harms.

Consequently, it is important that those creating and using AI in decision pathways understand the limitations of such technology, and have clear use cases. Sometimes this might involve a determination that an AI is not to be wholly relied upon as an autonomous decision for a particular application, but it is a tool to supplement a clinical decision made by a clinician (i.e., it might be regarded as a diagnostic tool or clinical support system). Understanding the respective roles of the HCP and AI to guide, inform or make a diagnosis is also critical from the perspective of medical devices regulation, since the intended purpose of the software dictates its eligibility to be placed on the market in the UK and may inform questions of liability and responsibility (14). This important question of how AI can aid, as opposed to replace doctors entirely, is therefore essential for further regulatory clarity on how liability and moral responsibility should be delineated.

As the participants agreed, moral and legal responsibility must remain with human experts. Even in cases where AI is fully autonomous, some humans will need to continue to oversee such decisions to detect malfunctioning and observe that the system continues to learn and adapt appropriately (1, 15–17). This is also the approach taken in other areas, such as in the regulation of autonomous vehicles, where Parliament (18, 19) and the Law Commission (20) have considered it legally and ethically inappropriate for high-risk AI to be fully automated (19).

### 4.3. Justice: equity and bias

Eradicating bias and assessing whether AI helps achieve healthcare equality is not just a question of discrimination but also of justice (10, p 225). As an ethical principle, justice can be interpreted as both being about utility (cost and risk analysis) and the interests of individual patients (21). One of the most prominent concerns in the AI field at present relates to bias and how it risks impacting those on the receiving end of AI system *decisions*. For example, the diversity of the training cohort can impact the working accuracy of AI and consequently, unjustly lead to only those it was trained on being able to reap its benefits. Likewise, the demographics of the training cohort may still impact trust in the system where the perception of potential bias can be just as harmful as actual bias. It is important that the values that are written into the algorithm are defensible or certain patients may be excluded (22).

Understanding the scalability of the model provides further nuance. The issue of human bias seeping into algorithms through biased data sets is also well-known as an issue of “big data”. However, not all AI holds the same risks meaning that a uniform approach will likely lead to some AI being subjected to disproportionate over-regulation which could result in patients being harmed through being barred access to these AI.

Moreover, bias can present itself in unexpected ways and will require the developers and HCPs training such systems to challenge their own assumptions about whether bias could arise. Being clear on the current and future uses of the AI at the outset is needed to better ensure that the AI will work as predicted in those specified contexts. Participants raised the issue of unknown bias which highlighted the importance of such discussions so that patients could be assured that unknown bias has been considered (22). These concerns of bias touch on ethical principles such as justice and non-maleficence because such principles consider differential treatment and the potential harm or injustice they may cause. From a legal perspective, national and supranational human rights such as the right to health (23, 24) and freedom from discrimination (23–25) also place obligations on State run institutions and actors (such as the NHS) to uphold “equal treatment” in healthcare (as well as other sectors).

### 4.4. Autonomy: transparency/oversight

Transparency is also crucial to the challenge of using AI in a meaningful way. It covers a wide range of issues such as trust and how to tackle misinformation. Participants considered the challenge of how to best communicate the risks and benefits of AI to patients and whether current data protection law initiatives that seek to build trust and ensure transparency are sufficient. This is because whilst data protection law stipulates that information must be provided to patients, the subsequent questions of how much and what specifically needs to be provided is open to interpretation, and is guided by best practice guidance and relevant court judgments (see next subsection).

This has also yet to be considered fully by the medical device regulator in their proposals for regulation of software as medical device (SaMDs) or artificial intelligence as medical device (AIaMDs) (11). Moreover, whilst data protection law indicates that relevant information must be provided (12, see Article 5(1)(a)), the justification for this stipulation is very broad with a danger that healthcare providers might “over-provide” information. Taking such an exceptional approach could have negative consequences if it leads to greater public concern than is justified, simply due to the involvement of AI. In considering what information should be provided, the participants suggested a practical approach by focusing on the priorities of different stakeholders. It was suggested that frontline HCPs are more likely to require information about the accuracy of outputs, for example information explaining why certain areas of a slide were flagged.

### 4.5. Autonomy: patient choice

Patient choice is a further legal and ethical consideration. From a legal perspective, patient choice holds paramount importance in upholding patients' rights to bodily integrity and bodily autonomy (26, 27). However, choice is a legally and ethically limited “right”; how samples are processed, what tests are conducted and what treatment options are made available is left to medical professionals to decide on the basis of their professional discretion, including their role in overseeing how NHS resources are spent. Nevertheless, the new territory of AI-human pathways begs the question of whether there is something so ethically and legally different about the benefits and risks that healthcare AI carries, that patients should have a choice on how or if at all, any part of the pathway is automated in their care.

Generally, patients will continue to have a right to refuse treatment if they distrust AI guided decision-making as the right extends to any reason for refusal given by a patient (27, 28). However, it is less clear if the right to refuse would stretch to refusing to have slides of samples taken from them to be analysed by AI. Traditionally, standards of care relating to diagnosis are ultimately the prerogative of those setting and implementing medical standards under the common law. However, such cases have entered murkier waters in recent years, where a handful of high court cases have sought to redraw the boundaries around the seminal tests for negligence by distinguishing between negligent treatment cases and negligent diagnosis and have tried to question if those tests apply to the latter (27, 29–31). For the time being, high courts have continued to apply these tests to both diagnosis and treatment decisions. This, combined with the short-to-medium future possibility of increasingly automated pathways, leads to some interesting considerations for developers and medical device manufacturers.

In cases of negligent diagnosis or treatment, the crucial legal question will presumably rest on whether AI amounts to a support tool or is an autonomous decision-maker (4, 32). Feedback from workshop participants highlighted the importance of humans staying in the loop as overseers and gatekeepers of appropriate diagnosis and treatment. For Cytosponge^TM^, the part of the pathway the workshop focused on, was largely diagnosis (interpreting the slides and feeding those results back to patients). Due to the changes in regulatory landscape noted above, it would therefore be important for developers to signpost that such technology is a support tool and not a decision-maker, and for care standards to also highlight the importance of medical staff using such AI reports as a mere guide within their wider decision-making expertise (33).

Given that the case of *Bolam* (34) remains an authority on both treatment and diagnosis, albeit with some caveats in certain instances (35), there is no reason to believe that a patient's right to refuse treatment would not apply where a patient objects to AI reading slides. However, the question of whether they could alternatively request human oversight or human performance as an alternative is legally challenging, and has been discussed in the literature (36). Under English common law, patients do not have a legally protected right to request treatment or request how their samples are processed (37). However, if such systems become increasingly automated, friction could develop with the UK GDPR's right to request human oversight where the diagnosis becomes solely automated (12, see Article 22). In such circumstances, a statutory right would have primacy, potentially creating exceptional treatment where AI is involved.

## 5. Conclusion

The ethical and legal considerations highlighted by stakeholders in the workshops will be relevant to decisions about AI automation and the extent of appropriate human involvement in almost all areas of healthcare. However, the specific content implications of the ethical and legal factors we identified will be highly context-specific and to address them will require multidisciplinary focus and engagement with patient groups. Medical and professional bodies are best placed to lead these discussions in relation to specific medical contexts. However, an exceptional approach to AI healthcare tools would be inappropriate and the comparison should be with existing (imperfect) practice and human decision making. Further research is needed on the application of specific existing regulations, in particular, Article 22 of the GDPR, which may have a powerful influence on acceptable forms of automation in practice in healthcare settings. Further work is also required to clarify how accountability should be assigned as AI automation develops along a clinical pathway, as current approaches suggest that medical practitioners will largely shoulder responsibility and liability for harm. Whether this is fair and reasonable needs assessment. Finally, all stakeholders should acknowledge the importance of patient trust and confidence which underpins whether AI automation will be accepted or rejected. It is vital that all stakeholders are proactive in engaging with patients and publics in order to address this issue in the healthcare context.

## Data Availability

The original contributions presented in the study are included in the article, further inquiries can be directed to the corresponding author/s.
